# Interpregnancy weight gain and childhood obesity: analysis of a UK population-based cohort

**DOI:** 10.1038/s41366-021-00979-z

**Published:** 2021-10-13

**Authors:** Nida Ziauddeen, Jonathan Y. Huang, Elizabeth Taylor, Paul J. Roderick, Keith M. Godfrey, Nisreen A. Alwan

**Affiliations:** 1 School of Primary Care, Population Sciences and Medical Education, Faculty of Medicine, University of Southampton, Southampton, UK; 2 NIHR Applied Research Collaboration Wessex, Southampton, UK; 3 Singapore Institute for Clinical Sciences (SICS), Agency for Science, Technology and Research (A*STAR), Singapore, Singapore; 4 NIHR Southampton Biomedical Research Centre, University of Southampton and University Hospital Southampton NHS Foundation Trust, Southampton, UK; 5 MRC Lifecourse Epidemiology Unit, University of Southampton, Southampton General Hospital, Southampton, UK

**Keywords:** Risk factors, Epidemiology

## Abstract

**Background:**

Maternal obesity increases the risk of adverse long-term health outcomes in mother and child including childhood obesity. We aimed to investigate the association between interpregnancy weight gain between first and second pregnancies and risk of overweight and obesity in the second child.

**Methods:**

We analysed the healthcare records of 4789 women in Hampshire, UK with their first two singleton live births within a population-based anonymised linked cohort of routine antenatal records (August 2004 and August 2014) with birth/early life data for their children. Measured maternal weight and reported height were recorded at the first antenatal appointment of each pregnancy. Measured child height and weight at 4–5 years were converted to age- and sex-adjusted body mass index (BMI z-score). Log-binomial regression was used to examine the association between maternal interpregnancy weight gain and risk of childhood overweight and obesity in the second child. This was analysed first in the whole sample and then stratified by baseline maternal BMI category.

**Results:**

The prevalence of overweight/obesity in the second child was 19.1% in women who remained weight stable, compared with 28.3% in women with ≥3 kg/m^2^ weight gain. Interpregnancy gain of ≥3 kg/m^2^ was associated with increased risk of childhood overweight/obesity (adjusted relative risk (95% CI) 1.17 (1.02–1.34)), with attenuation on adjusting for birthweight of the second child (1.08 (0.94–1.24)). In women within the normal weight range at first pregnancy, the risks of childhood obesity (≥95th centile) were increased with gains of 1–3 kg/m^2^ (1.74 (1.07–2.83)) and ≥3 kg/m^2^ (1.87 (1.18–3.01)).

**Conclusion:**

Children of mothers within the normal weight range in their first pregnancy who started their second pregnancy with a considerably higher weight were more likely to have obesity at 4–5 years. Supporting return to pre-pregnancy weight and limiting weight gain between pregnancies may achieve better long-term maternal and offspring outcomes.

## Introduction

The prevalence of obesity in women of reproductive age is rising worldwide and is seen in all populations regardless of income status [1]. Maternal obesity during pregnancy in England has shown a major increase over time, from 7.6% in 1989 to 15.6% in 2007 [2] to 22.2% in 2018/19 [3], with the rate of normal weight pregnancies decreasing from 65.6% in 2007 to 53.6% in 2007 [2] and 46.3% in 2018/19 [3]. Maternal obesity during pregnancy increases the risk of adverse pregnancy outcomes for both mother and child. It also increases the risk of long-term health problems in the child including obesity, cardiovascular disease and diabetes [4].

Pregnancy can alter a woman’s weight trajectory due to the risk of weight gain with childbearing for biological and behavioural reasons [5]. Weight gained during pregnancy is not always lost after delivery and thus pregnancy is a risk factor for overweight and obesity in women, which increases with additional pregnancies [6]. Analysis of data from the Danish Medical Birth Registry between 2004 and 2012 showed an increase in maternal BMI with each additional pregnancy [7]. Similarly, childbearing has been found to have a persistent long-term effect on adiposity in women in the UK with a progressive BMI increase observed from nulliparous women to multiparous women with four or more births [8]. A systematic review of 25 studies found that postpartum weight shows a continuous decrease until 12 months following which there is some evidence of increase in weight [5]. Weight retention post-partum is variable with women on average retaining 0.5 to 3 kg; however, a substantial number (12–20%) retain a considerable amount of weight (up to 17.7 kg) [9]. Interpregnancy weight loss in population-based cohorts has ranged from 11% [10] to 16% [11].

Birth registration data from England and Wales shows that 63% of women have two or more children (37% have two, 16% have three and 10% have four or more) [12]. An interpregnancy interval (interval between the birth of a child to the conception of the next child) of ≥36 months is associated with greater risk of starting a subsequent pregnancy at a higher weight [13]. Previous research has found an increased risk of gestational diabetes (GDM), caesarean section [14–16] and pre-eclampsia [15, 16] with interpregnancy weight gain particularly in women with healthy first pregnancy BMI (<25 kg/m^2^). Interpregnancy weight gain is associated with an increased risk of large-for-gestational age (LGA) birth [10, 11, 17], which, in turn, is associated with both childhood [18, 19] and adult obesity [20–22]. The mechanisms are unclear but the increase in adiposity on weight retention or gain postpartum may be a contributing factor to these associations.

Interpreting the findings from studies on maternal weight change is complicated by the fact that weight gain (e.g. amongst underweight) or loss (e.g. amongst overweight) may differ between individuals and across contexts. To address this, our large population-based study primarily focussed on non-underweight women who maintained or gained weight between their first two pregnancies. The aim was to investigate the association between maternal weight gain between the first and second singleton live birth pregnancies and the risk of overweight and obesity in the second child. As the effect of weight gain/retention may differ by maternal BMI at the start of the first pregnancy, we stratified the analyses by maternal BMI. To investigate potential mechanisms, we aimed to examine whether birthweight and postnatal factors such as breastfeeding accounted for the observed relationships.

## Methods

SLOPE (Studying Lifecourse Obesity PrEdictors) is a population-based anonymised linked cohort of prospectively collected routine maternal antenatal and birth records and child health records for all births registered at University Hospital Southampton (UHS), in the South of England, UK between January 2003 and April 2018. UHS is the regional centre for maternity care to residents in the city of Southampton and the surrounding areas of Hampshire. Child healthcare for the same area is provided by two community National Health Service (NHS) trusts; Solent and Southern Health. Thus, the antenatal and birth records (*n* = 83,481) were then linked to child health data from these two community NHS trusts (*n* = 74,770, 90% linked).

**Fig. 1:**
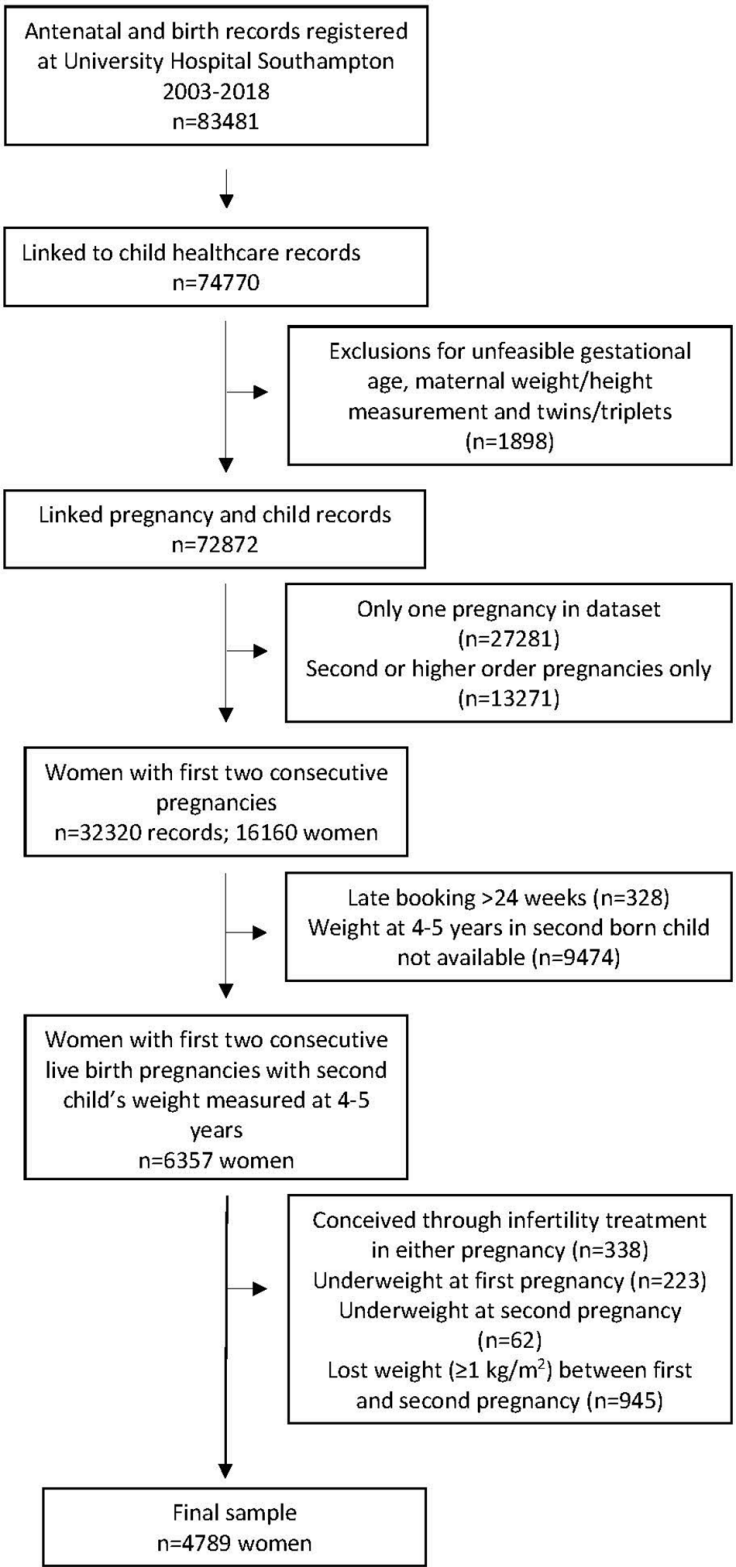
PRISMA flow diagram. Flowchart of the study including details of inclusion and exclusion criteria.

Records of women with their first two consecutive singleton live birth pregnancies that were successfully linked to child health data for the second child were included. Any woman who had a booking appointment at or after 24 weeks of pregnancy was excluded (Fig. 1). Only pregnancies with feasible gestational age (22–43 weeks), maternal weight and maternal height measurements were eligible for inclusion in this analysis (*n* = 6357). Women who conceived through infertility treatment in either pregnancies (*n* = 338), those who were underweight (BMI < 18.5 kg/m^2^) at first (*n* = 223) or second pregnancy (*n* = 62), and those who lost weight (≥1 kg/m^2^) between pregnancies (*n* = 945) were excluded from this analysis leaving data from 4789 women for analysis (75% of eligible sample). These exclusions were made to ensure a straightforward comparison between women who maintained and gained weight between pregnancies, and to reduce the potential for residual confounding due to unmeasured changes in health status which may differ between women who lost weight between pregnancies and others.

### Exposure assessment

Maternal weight in kilograms was routinely measured by a midwife at the first antenatal (booking) appointment of each pregnancy [23], which is recommended to take place ideally by 10 weeks gestation in the UK, according to the National Institute for Health and Care Excellence Guidelines [24]. Height was self-reported. BMI was calculated as weight/(height)^2^.

BMI at the start of the first pregnancy (baseline BMI) was categorised as normal weight (18.5–24.9 kg/m^2^), overweight (25.0– 29.9 kg/m^2^) and obesity (≥30 kg/m^2^). Change in BMI was calculated as the difference in BMI measured at the booking appointments of the first two consecutive live birth pregnancies for each woman. After excluding women who lost weight between their pregnancies (≥1 kg/m^2^), this change was categorised as weight stable (−1 to 1 kg/m^2^), moderate weight gain (1–3 kg/m^2^) (MWG) and substantial weight gain (≥3 kg/m^2^) (SWG).

### Outcome assessment

As part of the National Child Measurement Programme (NCMP), children in all state-maintained schools in England are measured by school nurses at Year R (4–5 years) and Year 6 (10–11 years) [25]. Only the measurement at 4–5 years was used in this analysis and children who did not have a weight and height measurement at 4–5 years were excluded (*n* = 9474). BMI was then calculated as weight/(height)^2^ and converted to age- and sex-adjusted BMI z-scores according to the UK 1990 growth reference charts [26]. The 85th percentile (z-score of +1.04) was used to specify the outcome of overweight and obesity and the 95^th^ percentile (z-score of +1.65) was used to specify the outcome of obesity [27, 28].

### Covariates

Maternal age (in years) was calculated from date of birth before extraction of the dataset to maintain anonymity. Highest maternal educational qualification was self-reported and categorised as secondary (GCSE) and under, college (A levels) and university degree or above. Self-reported ethnicity was recorded under 16 categories and condensed to White, Mixed, Asian, Black/African/Caribbean and Other. Categories of not asked and not stated were coded as missing. Smoking at booking was self-reported as current smoking or non-smoking. Non-smokers were further asked if they had ever smoked or had previously smoked and quit. This was categorised as stopped >12 months before conception, stopped <12 months before conception or stopped when pregnancy confirmed. Employment status was self-reported and categorised as employed, unemployed, in education, and not specified. In this population, an oral glucose tolerance test was used for screening for GDM in women with one or more risk factors (BMI > 30 kg/m^2^); GDM in previous pregnancy; previous baby weighing ≥4.5 kg; diabetes in parents or siblings and of Asian, African-Caribbean or Middle Eastern ethnicity) [29]. GDM diagnosis was then reported in the database. Interpregnancy interval was defined as the interval between the first live birth and conception of the second pregnancy. The difference in days between two consecutive live births was calculated and gestational age of the latter birth subtracted from this to derive the interpregnancy interval.

Birthweight (grams) was measured by healthcare professionals at birth as part of routine care. Gestational age was based on a dating ultrasound scan which routinely takes place between 10 and 13 weeks gestation [24]. Child sex was recorded at birth.

Breastfeeding status was reported at hospital discharge and during early life. The recording during early life was done differently by the two community NHS Trusts. One used NHS Read codes and thus was recorded at 10 days, 2 weeks, 6 weeks, 4 months and 9 months as breastfed, bottle-fed or breast and bottle fed; breastfeeding could be recorded at any or all of the time-points specified by the Read codes. The other Trust recorded breastfeeding at 56 days (8 weeks) as yes or no so there was no information on whether this was exclusive or partial breastfeeding. There was a small number (*n* < 10) of responses for breastfeeding for 4 months and 9 months at both pregnancies and no records for 8 weeks (for breastfeeding at first pregnancy only). Using all the information available, a breastfeeding variable was derived with categories of no breastfeeding, minimum 10 days and minimum 6 weeks. Minimum duration was chosen as there was no information how long breastfeeding continued beyond the point of the last record.

### Missing data

Of the women included, 83.9% of records had missing values for breastfeeding status at first pregnancy, 61.7% for breastfeeding status at second pregnancy, 3.8% for ethnicity and 0.3% on employment status. We imputed 85 datasets using multiple imputation via chained equations. The imputation models included the outcome and variables of analytical interest without missing values to impute the missing values for ethnicity, employment status and breastfeeding status in first and second pregnancy.

### Statistical analysis

Unadjusted comparisons were carried out using ANOVA for continuous variables and chi-square test for categorical variables.

The selection of covariates into the multivariable models were guided by a directed acyclic graph (DAG) (Supplementary Fig. 1) constructed using DAGitty [30]. Covariates comprised maternal age at first pregnancy, ethnicity, highest educational qualification, employment status at first pregnancy, smoking status at first and second pregnancy, first and second pregnancy gestational age at booking, first pregnancy BMI as measured at booking, GDM in first pregnancy, interpregnancy interval and breastfeeding status for first pregnancy (Model 1). To estimate the controlled direct effect of interpregnancy BMI gain, potential mediators were additionally adjusted for including GDM in second pregnancy, birthweight, gestational age at birth and breastfeeding status for second pregnancy (Model 2). Although child sex was included as an adjustment variable in the DAG, this was not included as the outcome is standardized for child sex.

The association between the maternal interpregnancy weight change with risk of childhood overweight and obesity in the second child was examined by fitting generalised linear models predicting each of the two binary outcomes (overweight or obesity) to categories of BMI gain (with stable BMI as the referent category) and covariates using a log link [31] (i.e. log-binomial regression). This was analysed first in the whole sample and then stratified by baseline maternal BMI category. A statistical significance level of 0.05 with 95% confidence intervals was used in the models.

Covariate adjustments for downstream consequences of exposures (e.g. mediators) has long been known to be a potential source of bias [32], particularly in obstetrics [33] and perinatal epidemiology [34] where adjustments for health indicators such as birthweight and gestational age are common and may lead to paradoxical findings. We investigated the sensitivity of our original models to such collider stratification bias by using inverse probability weighting to balance the distribution of exposure and mediators before any covariate adjustments. Weights were calculated by estimating separate propensity scores for the exposure and each mediator based on their respective confounders specified in the DAG and taking their inverse. Each subject was then weighted by the product of exposure and mediator weights in corresponding analyses. Under the strong assumption that all exposure- and mediator- outcome confounding is properly adjusted with no meaningful interactions, the resulting estimates correspond to the effect of interpregnancy weight change in a population where individuals all have similar likelihood of attaining observed mediator values. That is, if assumptions are fulfilled, such models can be used to estimate of the remaining effect of interpregnancy weight gain on second child overweight if interventions on pregnancy and birth outcomes could be taken.

All analyses were performed using Stata 15 [35].

### Ethical considerations

Data were anonymised by the data holders before being accessed by the research team. Ethics approval was granted by the University of Southampton Faculty of Medicine Ethics Committee (ID 24433) and Health Research Authority (HRA) (IRAS 242031).

## Results

Information on the first and second singleton live birth pregnancies and BMI of second child at 4–5 years was available for 4789 women. Of these, 42.7% women remained weight stable, 33.5% exhibited MWG (1–3 kg/m^2^) and 23.7% exhibited SWG (≥3 kg/m^2^). Mean maternal BMI at second pregnancy was 24.1 kg/m^2^ (standard deviation (SD) 4.1) in women who remained weight stable, 26.1 kg/m^2^ (SD 4.5) in women with MWG and 31.1 kg/m^2^ (SD 5.8) in women with SWG (Table 1). There was a slight increase in the proportion of women with overweight in the second pregnancy but the proportion with obesity nearly tripled from first (18.3%) to second (51.0%) pregnancy in women with SWG. Thirty percent of women who remained weight stable had overweight or obesity at second pregnancy compared to 50.6% with MWG and 88.2% with SWG.

**Table 1:** Maternal and birth characteristics categorised by maternal weight change from the first live birth pregnancy for the period of January 2003–September 2017, University Hospital Southampton NHS Foundation Trust, Hampshire, England.

	Weight stable (> −1 to <1 kg/m^2^)	Moderate weight gain (1–3 kg/m^2^)	Substantial weight gain (≥3 kg/m^2^)	p*
*N*	2047	1605	1137	
Maternal age at first pregnancy, years (mean ± SD)	27.1 ± 5.2	26.2 ± 5.3	23.7 ± 5.4	<0.001
Maternal age at second pregnancy, years (mean ± SD)	29.8 ± 5.2	29.1 ± 5.3	27.0 ± 5.5	<0.001
First pregnancy booking appointment, weeks (mean ± SD)	11.3 ± 2.5	11.4 ± 2.6	11.4 ± 2.8	0.78
Second pregnancy booking appointment, weeks (mean ± SD)	10.9 ± 2.3	11.2 ± 2.3	10.9 ± 2.5	0.002
Maternal BMI at first pregnancy booking, kg/m^2^ (mean ± SD)	24.0 ± 4.1	24.3 ± 4.4	26.0 ± 5.1	<0.001
Maternal BMI at second pregnancy booking, kg/m^2^ (mean ± SD)	24.1 ± 4.1	26.1 ± 4.5	31.1 ± 5.8	<0.001
Maternal BMI category at first pregnancy booking (%, 95% CI)				
Normal weight (18.5 to 24.9)	70.2 (68.2 to 72.2)	65.4 (63.0 to 67.7)	48.5 (45.6 to 51.5)	<0.001
Overweight (25.0 to 29.9)	20.9 (19.2 to 22.7)	23.9 (21.8 to 26.0)	33.2 (30.4 to 36.0)	
Obesity (≥30.0)	8.8 (7.6 to 10.2)	10.7 (9.2 to 12.3)	18.3 (16.1 to 20.7)	
Maternal BMI category at second pregnancy booking (%, 95% CI)				
Normal weight (18.5 to 24.9)	70.0 (67.9 to 71.9)	49.3 (46.9 to 51.8)	11.8 (10.0 to 13.8)	<0.001
Overweight (25.0 to 29.9)	20.7 (19.0 to 22.6)	33.8 (31.5 to 36.2)	37.2 (34.4 to 40.1)	
Obesity (≥30.0)	9.3 (8.1 to 10.6)	16.8 (15.0 to 18.7)	51.0 (48.1 to 54.0)	
Maternal smoking status at first pregnancy booking (%, 95% CI)				
Never smoked/quit	55.4 (53.2 to 57.6)	52.7 (50.2 to 55.2)	42.0 (39.1 to 44.9)	<0.001
Stopped >1 year before conceiving	14.9 (13.3 to 16.5)	13.5 (11.9 to 15.3)	9.8 (8.1 to 11.6)	
Stopped <1 year prior to conceiving	7.3 (6.2 to 8.5)	8.9 (7.6 to 10.4)	8.6 (7.1 to 10.4)	
Stopped when pregnancy confirmed	10.7 (9.4 to 12.2)	10.7 (9.2 to 12.3)	17.0 (14.8 to 19.3)	
Continued smoking	11.7 (10.4 to 13.2)	14.1 (12.5 to 15.9)	22.7 (20.3 to 25.2)	
Maternal smoking status at second pregnancy booking (%, 95% CI)				
Never smoked/quit	60.9 (58.7 to 63.0)	59.1 (56.7 to 61.5)	46.9 (43.9 to 49.8)	<0.001
Stopped >1 year before conceiving	20.1 (18.4 to 21.9)	18.3 (16.4 to 20.2)	16.4 (14.3 to 18.7)	
Stopped <1 year prior to conceiving	3.0 (2.3 to 3.9)	4.0 (3.1 to 5.1)	4.7 (3.6 to 6.2)	
Stopped when pregnancy confirmed	5.9 (4.9 to 7.0)	6.9 (5.7 to 8.3)	10.8 (9.1 to 12.7)	
Continued smoking	10.1 (8.8 to 11.4)	11.7 (10.1 to 13.3)	21.1 (18.8 to 23.6)	
Maternal education (%, 95% CI)				
Secondary (GCSE) or under	24.4 (22.6 to 26.3)	28.8 (26.6 to 31.1)	35.4 (32.7 to 38.3)	<0.001
College (A levels)	40.6 (38.5 to 42.8)	40.9 (38.5 to 43.4)	47.9 (45.0 to 50.9)	
University degree or above	34.9 (32.9 to 37.0)	30.3 (28.0 to 32.6)	16.6 (14.5 to 18.9)	
Maternal employment status at first pregnancy (%, 95% CI)				
Employed	89.0 (87.6 to 90.3)	84.6 (82.8 to 86.3)	74.1 (71.5 to 76.7)	<0.001
Unemployed	8.5 (7.4 to 9.8)	11.5 (9.9 to 13.1)	19.3 (17.0 to 21.7)	
In education	2.1 (1.5 to 2.8)	3.6 (2.7 to 4.6)	6.1 (4.8 to 7.6)	
Not specified	0.3 (0.1 to 0.7)	0.3 (0.1 to 0.8)	0.5 (0.2 to 1.1)	
Maternal employment status at second pregnancy (%, 95% CI)				
Employed	74.2 (72.3 to 76.1)	67.7 (65.3 to 69.9)	55.7 (52.7 to 58.6)	<0.001
Unemployed	24.4 (22.6 to 26.3)	30.3 (28.0 to 32.6)	42.3 (39.4 to 45.2)	
In education	0.7 (0.4 to 1.2)	1.2 (0.7 to 1.8)	1.2 (0.7 to 2.1)	
Not specified	0.6 (0.3 to 1.1)	0.8 (0.5 to 1.5)	0.8 (0.4 to 1.5)	
Ethnicity (%, 95% CI)				
White	90.9 (89.6 to 92.1)	87.7 (86.0 to 89.3)	87.5 (85.4 to 89.4)	<0.001
Mixed	0.6 (0.3 to 1.0)	0.9 (0.5 to 1.5)	1.7 (1.0 to 2.6)	
Asian	3.1 (2.4 to 4.0)	4.5 (3.5 to 5.6)	5.8 (4.5 to 7.3)	
Black/African/Caribbean	0.5 (0.3 to 1.0)	1.7 (1.2 to 2.5)	2.5 (1.6 to 3.5)	
Other	0.7 (0.4 to 1.2)	0.4 (0.2 to 0.9)	0.4 (0.1 to 1.0)	
Not specified	4.1 (3.3 to 5.1)	4.7 (3.7 to 5.8)	2.1 (1.4 to 3.1)	
Interpregnancy interval (median, IQR)	21.4 (14.4 to 31.4)	22.7 (13.8 to 33.1)	26.2 (15.6 to 40.5)	<0.001
Interpregnancy interval (%, 95% CI)				
0–11 months	16.7 (15.1 to 18.3)	18.4 (16.6 to 20.4)	16.7 (14.6 to 19.0)	<0.001
12–23 months	41.9 (39.7 go 44.0)	36.2 (33.8 to 38.6)	29.4 (26.7 to 32.1)	
24–35 months	24.2 (22.4 to 26.1)	24.6 (22.5 to 26.8)	23.5 (21.0 to 26.1)	
36 months or more	17.2 (15.6 to 19.0)	20.7 (18.8 to 22.8)	30.4 (27.8 to 33.2)	
Birthweight (second pregnancy), grams (mean ± SD)	3505 ± 501	3550 ± 515	3560 ± 547	0.004
Size at birth (second pregnancy)				
Small-for-gestational age	6.1 (5.0 to 7.1)	5.3 (4.3 to 6.5)	6.5 (5.1 to 8.1)	0.06
Appropriate-for-gestational age	80.8 (79.1 to 82.5)	80.4 (78.4 to 82.3)	76.9 (74.4 to 79.4)	
Large-for-gestational age	13.1 (11.7 to 14.7)	14.3 (12.6 to 16.1)	16.5 (14.4 to 18.8)	
BMI category at age 4–5 years (second pregnancy)				
Normal weight (<85th centile)	80.9 (79.1 to 82.6)	78.5 (76.4 to 80.5)	71.7 (69.0 to 74.3)	<0.001
Overweight (≥85th-<95th centile)	12.2 (10.8 to 13.7)	14.0 (12.4 to 15.8)	16.3 (14.2 to 18.5)	
Obesity (≥95th centile)	6.9 (5.9 to 8.1)	7.5 (6.2 to 8.9)	12.0 (10.2 to 14.1)	

*p values calculated using ANOVA for continuous and chi-square test for categorical variables.

Women with SWG were more likely to be younger, smokers, unemployed and of lower educational attainment and have longer interval between pregnancies compared to those who remained weight stable between pregnancies. Women with SWG were also more likely to have overweight and obesity at both first and second pregnancies.

**Fig. 2:**
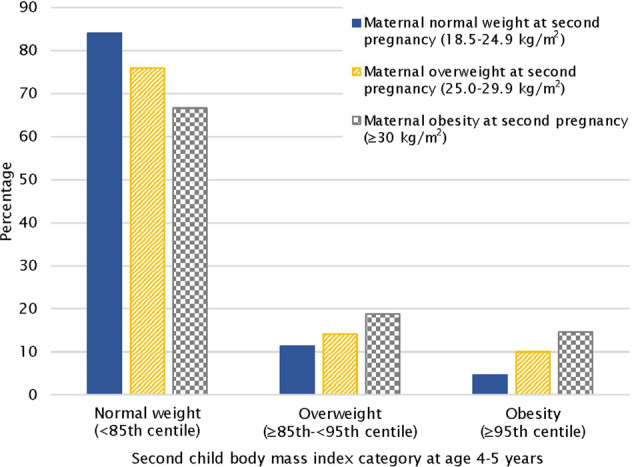
Prevalence of overweight and obesity in the second child at 4-5 years by maternal BMI category at second pregnancy. Prevalence of overweight and obesity by maternal BMI category.

The prevalence of overweight and obesity at 4–5 years in the second-born child increased from 15.9% in women who were normal weight at second pregnancy to 33.4% in women with obesity at the start of second pregnancy (Fig. 2). The prevalence of overweight and obesity in the second-born child increased from 19.1% in women who remained weight stable between pregnancies to 21.5% in women with MWG to 28.3% in women with SWG.

A higher proportion of second-born children of women with SWG had obesity (12.0%) compared to children of women who remained weight stable (6.9%) or with MWG (7.5%). Children of women with SWG were at increased risk of childhood overweight and obesity (≥95th centile) (adjusted relative risk (aRR) 1.17, 95% confidence interval (CI) 1.02–1.34) compared to remaining weight stable (Table 2). The relationship was attenuated on adjusting for birthweight, gestational age at birth, gestational diabetes in second pregnancy and breastfeeding status in second pregnancy (aRR 1.08, 95% CI 0.94–1.24). The attenuation was mainly from the adjustment for birthweight (Supplementary Table 1). This pattern for SWG was similar in the subgroup with obesity at the start of their first pregnancy (aRR 1.34, 95% CI 1.02–1.78, and aRR 1.25, 95% CI 0.94–1.64, respectively). A similar pattern was observed in women who were normal weight (aRR 1.16, 95% CI 0.93–1.45, and aRR 1.07, 95% CI 0.85–1.34) at the start of their first pregnancy. There was no evidence of association between MWG weight gain and childhood overweight and obesity. The association between SWG and childhood overweight and obesity persist when conducted using IPW (Supplementary Table 2).

**Table 2:** Associations between risk of overweight and obesity (85th centile) in the second child at age 4–5 years and change in maternal body mass index (BMI) between pregnancies as measured at the first antenatal visit of each pregnancy stratified by BMI category in the first pregnancy.

Maternal BMI change (categorised)		Full sample			Normal weight at first pregnancy			Overweight at first pregnancy			Obesity at first pregnancy		
		*n*, n of cases	Relative risk, (RR)*	95% CI	*n*, n of cases	RR*	95% CI	*n*, n of cases	RR^a^	95% CI	*n*, n of cases	RR^a^	95% CI
Weight stable (> −1 to <1 kg/m^2^)		2047, 391	Ref		1438, 220	Ref		428, 111	Ref		181, 60	Ref	
Moderate weight gain (1–3 kg/m^2^)	Unadjusted	1605, 345	1.13	0.99 to 1.28	1050, 188	1.17	0.98 to 1.40	383, 96	0.97	0.76 to 1.22	172, 61	1.07	0.80 to 1.43
	Model 1		1.06	0.92 to 1.21		1.15	0.95 to 1.38		0.94	0.74 to 1.20		1.04	0.75 to 1.43
	Model 2		1.01	0.89 to 1.16		1.10	0.91 to 1.32		0.93	0.74 to 1.17		1.00	0.74 to 1.37
Substantial weight gain (≥3 kg/m^2^)	Unadjusted	1137, 322	**1.48**	**1.30 to 1.69**	552, 124	**1.47**	**1.21 to 1.79**	377, 103	1.05	0.84 to 1.33	208, 91	**1.38**	**1.07 to 1.78**
	Model 1		**1.17**	**1.02 to 1.34**		1.16	0.93 to 1.45		0.94	0.73 to 1.22		**1.34**	**1.02 to 1.78**
	Model 2		1.08	0.94 to 1.24		1.07	0.85 to 1.34		0.94	0.74 to 1.19		1.25	0.94 to 1.64

Model 1: adjusted for maternal age at first pregnancy, ethnicity, highest educational qualification, smoking status at first and second pregnancy, employment status at first pregnancy, first and second pregnancy gestational age at booking appointment, baseline BMI, gestational diabetes in first pregnancy, interpregnancy interval and breastfeeding status for first pregnancy.

Model 2: adjusted for maternal age at first pregnancy, ethnicity, highest educational qualification, smoking status at first and second pregnancy, employment status at first pregnancy, first and second pregnancy gestational age at booking appointment, baseline BMI, gestational diabetes in second pregnancy, interpregnancy interval, birthweight, gestational age at birth and breastfeeding status for second pregnancy.

^a^Generalised linear model with log link and robust variance estimator used to derive RR

Bold values indicate statistical significant results.

Both MWG and SWG were associated with increased risk of childhood obesity (≥95th centile) only in women who were normal weight at first pregnancy (aRR 1.55, 95% CI 0.99–2.42 for MWG and aRR 1.74, 95% CI 1.11–2.73 for SWG) (Table 3). The relationship remained on adjusting for mediators of birthweight, gestational age at birth, gestational diabetes in second pregnancy and breastfeeding status in second pregnancy (aRR 1.74, 95% CI 1.07–2.83 for MWG and aRR 1.87, 95% CI 1.18–3.01 for SWG). There was no evidence of association between interpregnancy weight gain and childhood obesity in women with overweight or obesity at first pregnancy; however, the number of outcome events in this group were quite small. Analysis using IPW found the same association with childhood obesity in women who were normal weight at first pregnancy with SWG (Supplementary Table 3).

**Table 3:** Associations between risk of obesity (95th centile) in the second child at age 4–5 years and change in maternal body mass index (BMI) between pregnancies as measured at the first antenatal visit of each pregnancy stratified by BMI category in the first pregnancy.

Maternal BMI change (categorised)		Full sample			Normal weight at first pregnancy			Overweight at first pregnancy			Obesity at first pregnancy		
		*n*	Relative risk, (RR)^a^	95% CI	*n*	RR^a^	95% CI	*n*	RR^a^	95% CI	*n*	RR^a^	95% CI
Weight stable (> −1 to <1 kg/m^2^)		2047, 142	Ref		1438, 64	Ref		428, 47	Ref		181, 31	Ref	
Moderate weight gain (1–3 kg/m^2^)	Unadjusted	1605, 120	1.08	0.85 to 1.36	1050, 64	1.37	0.98 to 1.92	383, 34	0.81	0.53 to 1.23	172, 22	0.75	0.45 to 1.24
	Model 1		0.97	0.75 to 1.25		1.55	0.99 to 2.42		0.86	0.50 to 1.50		0.84	0.43 to 1.63
	Model 2		0.93	0.70 to 1.22		**1.74**	**1.07 to 2.83**		0.69	0.35 to 1.36		0.74	0.41 to 1.33
Substantial weight gain (≥3 kg/m^2^)	Unadjusted	1137, 137	**1.74**	**1.39 to 2.17**	552, 53	**2.16**	**1.52 to 3.06**	377, 41	0.99	0.67 to 1.47	208, 43	1.21	0.80 to 1.83
	Model 1		1.26	0.96 to 1.64		**1.74**	**1.11 to 2.73**		0.87	0.52 to 1.47		1.28	0.70 to 2.35
	Model 2		1.15	0.88 to 1.51		**1.87**	**1.18 to 3.01**		0.81	0.51 to 1.30		1.15	0.69 to 1.94

Model 1: adjusted for maternal age at first pregnancy, ethnicity, highest educational qualification, smoking status at first and second pregnancy, employment status at first pregnancy, first and second pregnancy gestational age at booking, baseline BMI, gestational diabetes in first pregnancy, interpregnancy interval and breastfeeding status for first pregnancy.

Model 2: adjusted for maternal age at first pregnancy, ethnicity, highest educational qualification, smoking status at first and second pregnancy, employment status at first pregnancy, first and second pregnancy gestational age at booking, baseline BMI, gestational diabetes in second pregnancy, interpregnancy interval, birthweight, gestational age at birth and breastfeeding status for second pregnancy.

^a^Generalised linear model with log link and robust variance estimator used to derive RR.

Bold values indicate statistical significant results.

Mean weight loss in women who lost weight (≤ −1 kg/m^2^) was 2.4 kg (SD 1.6). In fully adjusted models, the risk of child obesity were lower in mothers who lost weight (≤ −1 kg/m^2^) between pregnancies having overweight (aRR 0.69, 95% CI 0.38–1.23) or obesity (aRR 0.92, 95% CI 0.55–1.55) at their first pregnancy. However, the numbers of mothers in these groups were modest (304 and 200 women with overweight and obesitye respectively at first pregnancy) and the confidence intervals wide (Supplementary Table 4).

## Discussion

In this study sample, nearly a quarter of women gained ≥3 kg/m^2^ between their first and second pregnancies. Children of mothers within the normal weight range and with obesity who started their second pregnancy having gained ≥3 kg/m^2^ from their first pregnancy were more likely to have overweight or obesity (≥85th centile) at the start of primary school; this association was attenuated by accounting for birthweight; likely to be on the causal pathway between interpregnancy BMI gain and offspring obesity. Children of normal weight women who gained weight (1–3 and ≥3 kg/m^2^) from their first pregnancy were more likely to have obesity (≥95th centile) at the start of primary school.

The risk of overweight and obesity in the second child with interpregnancy weight gain was attenuated on adjusting for second pregnancy/birth factors. Maternal overweight and obesity is an established risk factor for GDM, higher birthweight and lower rates of breastfeeding [36], all of which are risk factors for childhood overweight and obesity [37–39]. Weight gain between pregnancies is associated with increased risk of GDM and LGA [14–16], particularly in normal weight women [14, 15]. Previous research examining the association between interpregnancy weight gain and LGA birth showed that women with overweight and obesity who dropped BMI category by their second pregnancy remained at an increased risk but had a lower risk compared to women whose BMI category increased between pregnancies [40]. Offspring of women with overweight and obesity are already at increased risk of childhood obesity and it is possible that the weight change in this subgroup was not large enough to detect a further increase in risk particularly in subgroups with small sample size. Greater efforts on primary prevention of preconception obesity in women of childbearing age are needed. However, it is important that any support policies fully recognise the wider social, environmental, economic and commercial determinants of obesity and avoid any implication of blame on mothers for their children’s overweight. Additionally, more effective weight loss measures for women with obesity and support for normal weight women to return to pre-pregnancy weight in the interpregnancy period are needed. Weight loss between pregnancies increases the risk of SGA but reduces the risk of LGA and GDM [14] and may be beneficial in protecting against childhood obesity.

The burden of maternal obesity, a key risk factor for childhood overweight and obesity, is growing and a need to focus on the preconception period has been highlighted to attempt to reverse the cycle and trans-generational effect of maternal obesity. Findings from this study are in line with findings from two previous studies. Analysis of 714 mother-child pairs from a population-based cohort in Australia showed that second-born offspring of women who gained ≥4 kg/m^2^ between their first and second pregnancy were at higher risk of overweight and obesity than those of women who remained weight stable (≥ −1 and <1 kg/m^2^) between pregnancies [41]. However, all measurements were self-reported by the mother, the response rate to the survey was low (34%), the proportion gaining ≥4 kg/m^2^ was small (38 women) and the analysis only adjusted for maternal factors before the birth of the second child. The proportion of women who remained weight stable in the Australian cohort (44.8%) was comparable to this cohort (42.7%). The other study utilised a population-based linked cohort in Scotland and found that maternal weight gain of ≥10% between pregnancies was associated with increased risk of overweight and obesity in the second-born child compared to remaining weight stable (±3%) [42]. However, only 44% of 59,975 records were successfully linked and 5863 women had more than one pregnancy in the linked dataset.

There is increasing evidence of the importance of the preconception and pregnancy periods on long-term health. A suggested approach to improving preconception health is to promote health of the population more broadly with targeting of women and partners planning a pregnancy [43–45]. It is important to engage with women during the interpregnancy period to optimise their and their children’s health and address the barriers against healthy behaviours as this is also the preconception period for the next pregnancy. The interpregnancy period provides an excellent opportunity to focus on preconception and family health due to the relatively intensive contact with health and care professionals. The feasibility and effectiveness of utilising existing contact time with healthcare professionals during the postpartum period to support maternal health needs to be explored. Mothers may also benefit from mutual support groups. Weight management issues tend to be greater in more disadvantaged mothers so the most effective strategies for such mothers needs to be identified to reduce social inequality in subsequent maternal, birth and child outcomes. As weight gain usually occurs in combination with other risk factors particularly in socioeconomically disadvantaged groups and women at this stage have competing priorities and time demands, a holistic approach taking all this into account should be considered.

### Strengths and limitations

The study sample is from a relatively large population-based cohort including women from all socioeconomic and ethnic backgrounds delivering at a large maternity centre in Southampton, UK, which is representative of the regional population. According to the UK Department of Communities and Local Government English indices of deprivation report, Southampton is more deprived than average, with the situation having worsened between 2010 and 2015 [46]. However, about half of the women included in this analysis reside in the rest of Hampshire (the region where Southampton is situated), which is less deprived. Our sample was 89% White, which is comparable to the 2011 England and Wales population census of 86% White [47]. The prevalence of childhood overweight and obesity in this linked sample is comparable to the average for the city of Southampton. The analysis was adjusted for several key confounders that were reasonably complete. Both the maternal weight (used to calculate exposure) and child height and weight (used to define outcome) were objectively measured by healthcare professionals.

An important limitation was the lack of information on gestational weight gain during pregnancy and paternal characteristics/behaviour, which may be mediators in the association between maternal interpregnancy weight gain and childhood overweight and obesity [48]. Breastfeeding initiation and duration after first pregnancy can influence post-partum weight and breastfeeding after the second pregnancy can influence the risk of overweight and obesity in the second child but information on breastfeeding was only recorded in a sixth of first and in little over a third of the second pregnancies so needed to be imputed. Women who had their first booking appointment later into the pregnancy (more than 24 weeks) were excluded from the analysis to ensure comparability of weight measurements between pregnancies. Women who lost weight were also excluded from this analysis to ensure comparability and to reduce the potential for residual confounding. Most of the confounding factors which were accounted for in the analysis were self-reported. Families had to stay in the area to remain under the care of the NHS Trust so data on further pregnancies or outcome data for the child was not available if the family moved outside of the area. Other factors contributing to missing outcome data potentially include changes in recording practices, not attending state school, or the child NHS number (required for linkage) was not recorded with the measurement.

In conclusion, one in five women had a BMI gain ≥3 kg/m^2^ between their first and second pregnancies. Children of mothers within the normal weight range and with obesity who started their second pregnancy with a considerably higher weight than their first were more likely to have overweight/obesity at the start of primary school; but this could be explained by gestational diabetes and breastfeeding status in the second pregnancy, birthweight and gestational age at birth. Children of normal weight women who gained weight between pregnancies were more likely to hav obesity at the start of primary school. The interpregnancy period between two pregnancies is a preconception intervention opportunity for subsequent pregnancies as women and their families have intensive contact with healthcare professionals after birth of a child. There is a need to support return to pre-pregnancy weight in normal weight women and weight loss in women with overweight and obesity.

## Supplementary information

Supplementary Figure 1

Supplementary Tables
