# Supplementary figures and images for: Interpregnancy weight gain and childhood obesity: analysis of a UK population-based cohort
Source: Int J Obes (Lond). 2021 Oct 13;46(1):211–9. doi: 10.1038/s41366-021-00979-z (PMC8748200; doi:10.1038/s41366-021-00979-z)

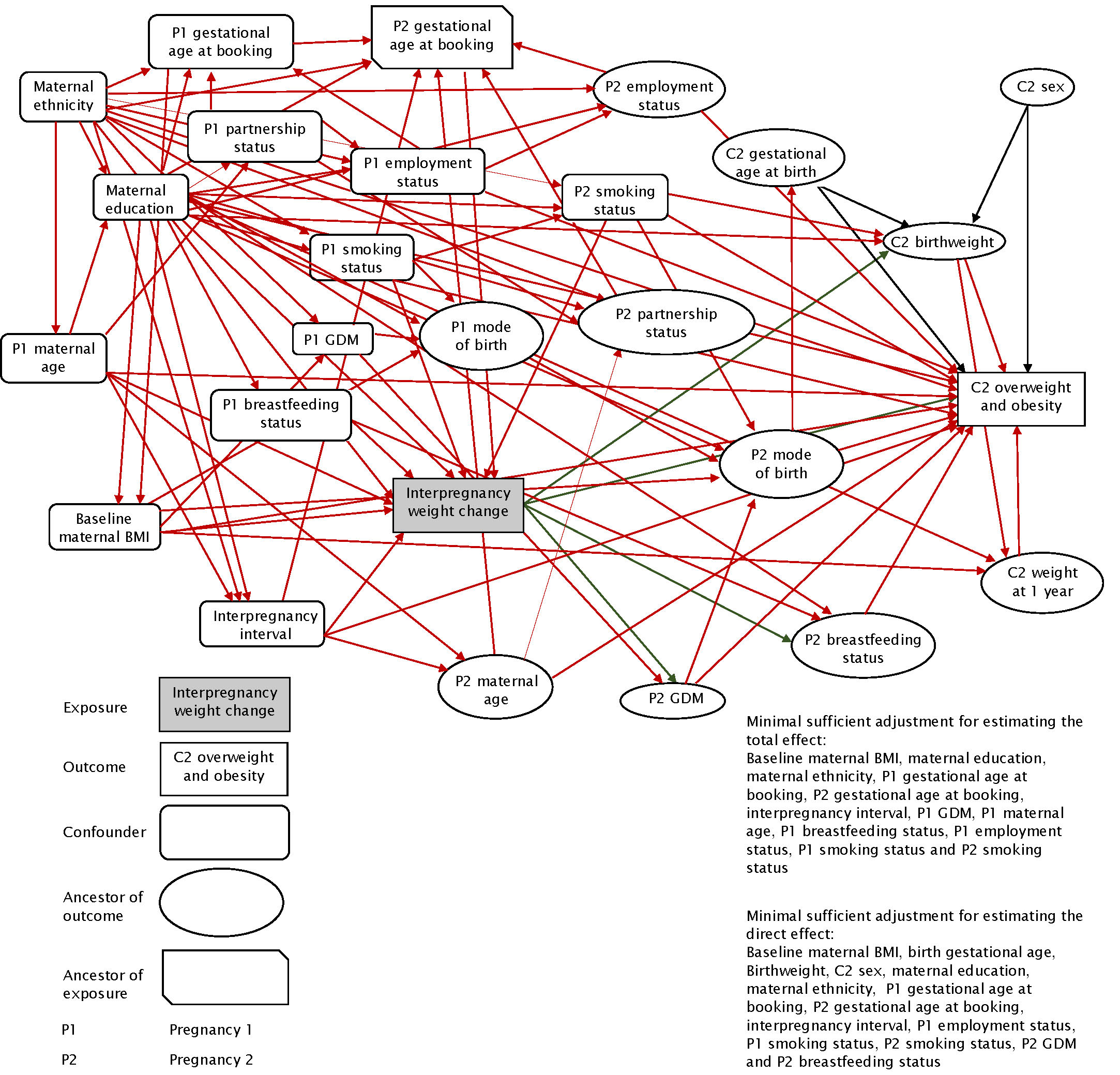

Supplement: Supplementary file 1 — Supplementary Figure 1 [file 41366_2021_979_MOESM1_ESM.jpg]
