# Supplementary material for: Interpregnancy weight gain and childhood obesity: analysis of a UK population-based cohort
Source: Int J Obes (Lond). 2021 Oct 13;46(1):211–9. doi: 10.1038/s41366-021-00979-z (PMC8748200; doi:10.1038/s41366-021-00979-z)
Supplement: Supplementary file 2 — Supplementary Tables [file 41366_2021_979_MOESM2_ESM.docx]

**Supplementary table legends**

Supplementary Table 1: Mediator stratified models for the associations between risk of overweight and obesity (85^th^ centile) in the second child at age 4-5 years and change in maternal body mass index (BMI) between pregnancies as measured at the first antenatal visit of each pregnancy stratified by BMI category

Supplementary table 2: Associations between risk of overweight and obesity (85^th^ centile) in the second child at age 4-5 years and change in maternal body mass index (BMI) between pregnancies as measured at the first antenatal visit of each pregnancy stratified by BMI category in the first pregnancy (using IPW)

Supplementary table 3: Associations between risk of overweight and obesity (95^th^ centile) in the second child at age 4-5 years and change in maternal body mass index (BMI) between pregnancies as measured at the first antenatal visit of each pregnancy stratified by BMI category in the first pregnancy (using IPW)

Supplementary Table 4: Associations between risk of overweight and obesity (85^th^ centile) and obesity (95^th^ centile) in the second child at age 4-5 years in women who lost weight (≤-1 kg/m^2^) between pregnancies as measured at the first antenatal visit of each pregnancy stratified by BMI category in the first pregnancy

Supplementary Table 1: Mediator stratified models for the associations between risk of overweight and obesity (85^th^ centile) in the second child at age 4-5 years and change in maternal body mass index (BMI) between pregnancies as measured at the first antenatal visit of each pregnancy stratified by BMI category

| Maternal BMI change (categorised) |  | Full sample | | | Normal weight at first pregnancy | | | Overweight at first pregnancy | | | Obese at first pregnancy | | |
| --- | --- | --- | --- | --- | --- | --- | --- | --- | --- | --- | --- | --- | --- |
|  |  | n, n of cases | Relative risk,  (RR)* | 95% CI | n, n of cases | RR* | 95% CI | n, n of cases | RR* | 95% CI | n, n of cases | RR* | 95% CI |
| Weight stable (>-1 to <1 kg/m^2^) |  | 2047, 391 | Ref |  | 1438, 220 | Ref |  | 428, 111 | Ref |  | 181, 60 | Ref |  |
| Moderate weight gain  (1-3 kg/m^2^) | Model 1 | 1605, 345 | 1.13 | 0.99 to 1.28 | 1050, 188 | 1.17 | 0.98 to 1.40 | 383, 96 | 0.97 | 0.76 to 1.22 | 172, 61 | 1.07 | 0.80 to 1.43 |
|  | Model 2a |  | 1.06 | 0.93 to 1.21 |  | 1.15 | 0.95 to 1.38 |  | 0.94 | 0.74 to 1.20 |  | 1.05 | 0.76 to 1.43 |
|  | Model 2b |  | 1.06 | 0.93 to 1.21 |  | 1.14 | 0.95 to 1.38 |  | 0.94 | 0.74 to 1.20 |  | 1.05 | 0.76 to 1.43 |
|  | Model 2c |  | 1.06 | 0.93 to 1.21 |  | 1.14 | 0.95 to 1.38 |  | 0.94 | 0.74 to 1.20 |  | 1.05 | 0.76 to 1.44 |
|  | Model 2d |  | 1.06 | 0.93 to 1.21 |  | 1.14 | 0.94 to 1.37 |  | 0.93 | 0.73 to 1.19 |  | 1.05 | 0.76 to 1.44 |
|  | Model 2e |  | 1.02 | 0.89 to 1.16 |  | 1.11 | 0.92 to 1.33 |  | 0.93 | 0.74 to 1.17 |  | 1.00 | 0.74 to 1.36 |
|  | Model 2 |  | 1.01 | 0.89 to 1.16 |  | 1.10 | 0.91 to 1.32 |  | 0.93 | 0.74 to 1.17 |  | 1.00 | 0.74 to 1.37 |
| Substantial weight gain  (≥3 kg/m^2^) | Model 1 | 1137, 322 | **1.48** | **1.30 to 1.69** | 552, 124 | **1.47** | **1.21 to 1.79** | 377, 103 | 1.05 | 0.84 to 1.33 | 208, 91 | **1.38** | **1.07 to 1.78** |
|  | Model 2a |  | **1.16** | **1.01 to 1.34** |  | 1.15 | 0.92 to 1.43 |  | 0.94 | 0.73 to 1.22 |  | **1.34** | **1.02 to 1.77** |
|  | Model 2b |  | **1.16** | **1.01 to 1.34** |  | 1.16 | 0.93 to 1.43 |  | 0.94 | 0.73 to 1.22 |  | **1.34** | **1.01 to 1.77** |
|  | Model 2c |  | **1.16** | **1.01 to 1.33** |  | 1.14 | 0.92 to 1.43 |  | 0.94 | 0.72 to 1.22 |  | **1.34** | **1.02 to 1.78** |
|  | Model 2d |  | **1.16** | **1.01 to 1.33** |  | 1.14 | 0.91 to 1.42 |  | 0.94 | 0.73 to 1.21 |  | **1.35** | **1.02 to 1.79** |
|  | Model 2e |  | 1.09 | 0.95 to 1.25 |  | 1.07 | 0.85 to 1.34 |  | 0.95 | 0.75 to 1.20 |  | 1.25 | 0.95 to 1.64 |
|  | Model 2 |  | 1.08 | 0.94 to 1.24 |  | 1.07 | 0.85 to 1.34 |  | 0.94 | 0.74 to 1.19 |  | 1.25 | 0.94 to 1.64 |

*Generalised linear model with log link and robust variance estimator used to derive RR

Model 1: adjusted for maternal age at first pregnancy, ethnicity, highest educational qualification, smoking status at first and second pregnancy, employment status at first pregnancy, first and second pregnancy gestational age at booking, baseline BMI, gestational diabetes in first pregnancy, interpregnancy interval and breastfeeding status for first pregnancy

Model 2a: adjusted for maternal age at first pregnancy, ethnicity, highest educational qualification, smoking status at first and second pregnancy, employment status at first pregnancy, first and second pregnancy gestational age at booking, baseline BMI and interpregnancy interval

Model 2b: Model 2a plus gestational diabetes in second pregnancy,

Model 2c: Model 2a plus breastfeeding status for second pregnancy

Model 2d: Model 2a plus gestational age at birth

Model 2e: Model 2a plus birthweight and gestational age at birth

Model 2: adjusted for maternal age at first pregnancy, ethnicity, highest educational qualification, smoking status at first and second pregnancy, employment status at first pregnancy, first and second pregnancy gestational age at booking, baseline BMI, gestational diabetes in second pregnancy, interpregnancy interval, birthweight, gestational age at birth and breastfeeding status for second pregnancy

Supplementary Table 2: Associations between risk of overweight and obesity (85^th^ centile) in the second child at age 4-5 years and change in maternal body mass index (BMI) between pregnancies as measured at the first antenatal visit of each pregnancy stratified by BMI category in the first pregnancy (using IPW)

| Maternal BMI change (categorised) |  | Full sample | | | Normal weight at first pregnancy | | | Overweight at first pregnancy | | | Obese at first pregnancy | | |
| --- | --- | --- | --- | --- | --- | --- | --- | --- | --- | --- | --- | --- | --- |
|  |  | n | Relative risk,  (RR)* | 95% CI | n | RR* | 95% CI | n | RR* | 95% CI | n | RR* | 95% CI |
| Weight stable (>-1 to <1 kg/m^2^) |  | 2047, 391 | Ref |  | 1438, 220 | Ref |  | 428, 111 | Ref |  | 181, 60 | Ref |  |
| Moderate weight gain  (1-3 kg/m^2^) | Unadjusted | 1605, 345 | 1.13 | 0.99 to 1.28 | 1050, 188 | 1.17 | 0.98 to 1.40 | 383, 96 | 0.97 | 0.76 to 1.22 | 172, 61 | 1.07 | 0.80 to 1.43 |
|  | Model 1 |  | 0.95 | 0.83 to 1.09 |  | 1.06 | 0.88 to 1.27 |  | 0.92 | 0.72 to 1.17 |  | 1.01 | 0.75 to 1.36 |
|  | Model 2 |  | 0.92 | 0.81 to 1.06 |  | 1.03 | 0.86 to 1.24 |  | 0.88 | 0.69 to 1.12 |  | 0.99 | 0.73 to 1.33 |
| Substantial weight gain  (≥3 kg/m^2^) | Unadjusted | 1137, 322 | **1.48** | **1.30 to 1.69** | 552, 124 | **1.47** | **1.21 to 1.79** | 377, 103 | 1.05 | 0.84 to 1.33 | 208, 91 | **1.38** | **1.07 to 1.78** |
|  | Model 1 |  | **1.27** | **1.11 to 1.45** |  | **1.34** | **1.10 to 1.64** |  | 1.00 | 0.79 to 1.27 |  | **1.32** | **1.01 to 1.72** |
|  | Model 2 |  | **1.23** | **1.08 to 1.41** |  | **1.31** | **1.07 to 1.60** |  | 0.96 | 0.76 to 1.22 |  | 1.30 | 1.00 to 1.69 |

Model 1: adjusted for maternal age at first pregnancy, ethnicity, highest educational qualification, smoking status at first and second pregnancy, employment status at first pregnancy, baseline BMI, gestational diabetes in first pregnancy, interpregnancy interval and breastfeeding status for first pregnancy

Model 2: adjusted for maternal age at first pregnancy, ethnicity, highest educational qualification, smoking status at first and second pregnancy, employment status at first pregnancy, baseline BMI, gestational diabetes in second pregnancy, interpregnancy interval, birthweight, gestational age at birth and breastfeeding status for second pregnancy

Supplementary Table 3: Associations between risk of obesity (95^th^ centile) in the second child at age 4-5 years and change in maternal body mass index (BMI) between pregnancies as measured at the first antenatal visit of each pregnancy stratified by BMI category in the first pregnancy (using IPW)

| Maternal BMI change (categorised) |  | Full sample | | | Normal weight at first pregnancy | | | Overweight at first pregnancy | | | Obese at first pregnancy | | |
| --- | --- | --- | --- | --- | --- | --- | --- | --- | --- | --- | --- | --- | --- |
|  |  | n | Relative risk,  (RR)* | 95% CI | n | RR* | 95% CI | n | RR* | 95% CI | n | RR* | 95% CI |
| Weight stable (>-1 to <1 kg/m^2^) |  | 2047, 142 | Ref |  | 1438, 64 | Ref |  | 428, 47 | Ref |  | 181, 31 | Ref |  |
| Moderate weight gain  (1-3 kg/m^2^) | Unadjusted | 1605, 120 | 1.08 | 0.85 to 1.36 | 1050, 64 | 1.37 | 0.98 to 1.92 | 383, 34 | 0.81 | 0.53 to 1.23 | 172, 22 | 0.75 | 0.45 to 1.24 |
|  | Model 1 |  | 0.85 | 0.66 to 1.09 |  | 1.24 | 0.88 to 1.75 |  | 0.73 | 0.47 to 1.12 |  | 0.71 | 0.42 to 1.20 |
|  | Model 2 |  | 0.82 | 0.64 to 1.05 |  | 1.21 | 0.86 to 1.72 |  | 0.70 | 0.45 to 1.07 |  | 0.68 | 0.40 to 1.15 |
| Substantial weight gain  (≥3 kg/m^2^) | Unadjusted | 1137, 137 | **1.74** | **1.39 to 2.17** | 552, 53 | **2.16** | **1.52 to 3.06** | 377, 41 | 0.99 | 0.67 to 1.47 | 208, 43 | 1.21 | 0.80 to 1.83 |
|  | Model 1 |  | **1.40** | **1.10 to 1.76** |  | **1.97** | **1.38 to 2.83** |  | 0.91 | 0.60 to 1.36 |  | 1.18 | 0.76 to 1.83 |
|  | Model 2 |  | **1.36** | **1.07 to 1.71** |  | **1.94** | **1.35 to 2.79** |  | 0.85 | 0.57 to 1.28 |  | 1.16 | 0.75 to 1.79 |

Model 1: adjusted for maternal age at first pregnancy, ethnicity, highest educational qualification, smoking status at first and second pregnancy, employment status at first pregnancy, first and second pregnancy gestational age at booking, baseline BMI, gestational diabetes in first pregnancy, interpregnancy interval and breastfeeding status for first pregnancy

Model 2: adjusted for maternal age at first pregnancy, ethnicity, highest educational qualification, smoking status at first and second pregnancy, employment status at first pregnancy, first and second pregnancy gestational age at booking, baseline BMI, gestational diabetes in second pregnancy, interpregnancy interval, birthweight, gestational age at birth and breastfeeding status for second pregnancy

Supplementary Table 4: Associations between risk of overweight and obesity (85^th^ centile) and obesity (95^th^ centile) in the second child at age 4-5 years in women who lost weight (≤-1 kg/m^2^) between pregnancies as measured at the first antenatal visit of each pregnancy stratified by BMI category in the first pregnancy

| Outcome |  | Full sample | | | Normal weight at first pregnancy | | | Overweight at first pregnancy | | | Obese at first pregnancy | | |
| --- | --- | --- | --- | --- | --- | --- | --- | --- | --- | --- | --- | --- | --- |
|  |  | n, n of cases | Relative risk,  (RR)* | 95% CI | n, n of cases | RR* | 95% CI | n, n of cases | RR* | 95% CI | n, n of cases | RR* | 95% CI |
| Overweight and obesity (≥85^th^ centile) | Unadjusted | 945, 221 | **1.22** | **1.06 to 1.42** | 441, 70 | 1.04 | 0.81 to 1.33 | 304, 77 | 0.98 | 0.76 to 1.26 | 200, 74 | 1.12 | 0.85 to 1.47 |
|  | Model 1 |  | 1.01 | 0.87 to 1.18 |  | 0.88 | 0.67 to 1.15 |  | 0.93 | 0.71 to 1.21 |  | 1.11 | 0.82 to 1.48 |
|  | Model 2 |  | 1.00 | 0.86 to 1.17 |  | 0.89 | 0.69 to 1.16 |  | 0.87 | 0.66 to 1.15 |  | 1.08 | 0.81 to 1.43 |
| Obesity (≥95^th^ centile) | Unadjusted | 945, 89 | 1.36 | 1.05 to 1.75 | 441, 24 | 1.22 | 0.77 to 1.93 | 304, 28 | 0.84 | 0.54 to 1.31 | 200, 37 | 1.08 | 0.70 to 1.67 |
|  | Model 1 |  | 1.01 | 0.75 to 1.37 |  | 1.20 | 0.70 to 2.05 |  | 0.70 | 0.39 to 1.27 |  | 1.04 | 0.59 to 1.83 |
|  | Model 2 |  | 1.01 | 0.75 to 1.38 |  | 1.50 | 0.81to 2.78 |  | 0.69 | 0.38 to 1.23 |  | 0.92 | 0.55 to 1.55 |

*Generalised linear model with log link and robust variance estimator used to derive RR

Model 1: adjusted for maternal age at first pregnancy, ethnicity, highest educational qualification, smoking status at first and second pregnancy, employment status at first pregnancy, first and second pregnancy gestational age at booking, baseline BMI, gestational diabetes in first pregnancy, interpregnancy interval and breastfeeding status for first pregnancy

Model 2: adjusted for maternal age at first pregnancy, ethnicity, highest educational qualification, smoking status at first and second pregnancy, employment status at first pregnancy, first and second pregnancy gestational age at booking, baseline BMI, gestational diabetes in second pregnancy, interpregnancy interval, birthweight, gestational age at birth and breastfeeding status for second pregnancy
